# Regulation of P-Glycoprotein in the Brain

**DOI:** 10.3390/ijms232314667

**Published:** 2022-11-24

**Authors:** Amanda B. Chai, Richard Callaghan, Ingrid C. Gelissen

**Affiliations:** 1 School of Pharmacy, Faculty of Medicine and Health, University of Sydney, Sydney, NSW 2006, Australia; 2 School of Biomedical Sciences, Faculty of Biological Sciences, University of Leeds, Leeds LS2 9JT, UK

**Keywords:** P-glycoprotein, ABCB1, blood–brain barrier, transcriptional regulation, post-transcriptional regulation, post-translational regulation

## Abstract

Maintenance of the tightly regulated homeostatic environment of the brain is facilitated by the blood–brain barrier (BBB). P-glycoprotein (P-gp), an ATP-binding cassette transporter, is expressed on the luminal surface of the endothelial cells in the BBB, and actively exports a wide variety of substrates to limit exposure of the vulnerable brain environment to waste buildup and neurotoxic compounds. Downregulation of P-gp expression and activity at the BBB have been reported with ageing and in neurodegenerative diseases. Upregulation of P-gp at the BBB contributes to poor therapeutic outcomes due to altered pharmacokinetics of CNS-acting drugs. The regulation of P-gp is highly complex, but unravelling the mechanisms involved may help the development of novel and nuanced strategies to modulate P-gp expression for therapeutic benefit. This review summarises the current understanding of P-gp regulation in the brain, encompassing the transcriptional, post-transcriptional and post-translational mechanisms that have been identified to affect P-gp expression and transport activity.

## 1. Introduction

The blood–brain barrier (BBB) serves as a dynamic and selective interface separating the central nervous system (CNS) from the periphery. Homeostasis within the CNS is maintained via coordination of physical, metabolic and transport-mediated mechanisms that carefully control the counter-directional transport of nutrients and waste products from the brain [1]. One of the key transport proteins expressed on the luminal membrane of the capillary endothelial cells that constitute the BBB is P-glycoprotein (P-gp), also referred to as ATP-binding cassette subfamily B member 1 (ABCB1) or multi-drug resistance protein 1 (MDR1) [2]. P-gp serves a protective function, utilising the energy from ATP hydrolysis to actively export a wide array of structurally diverse endogenous and exogenous substrates into the periphery [2]. P-gp contributes to BBB integrity, however, its involvement in neurological, neuroinflammatory and neurodegenerative conditions including Alzheimer’s disease and epilepsy, is growing in recognition [3]. Accordingly, increasing attention has been cast on the potential for modulating P-gp expression and/or activity for therapeutic benefit [4]. Hence, unravelling the regulatory mechanisms of P-gp has the potential to enhance our understanding of disease mechanisms and facilitate the discovery of new therapeutic targets.

**Figure 1:**
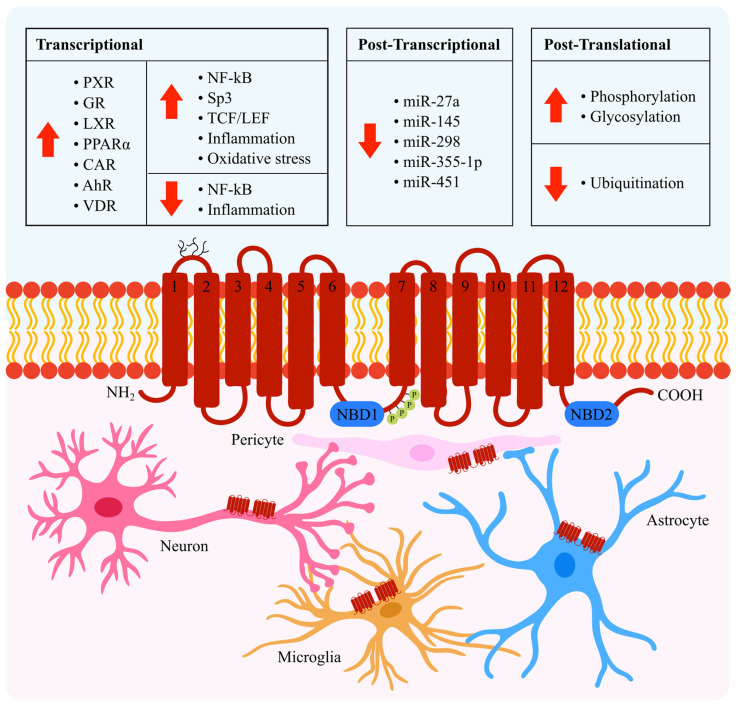
Structure, location and regulation of P-gp in the brain. The P-gp protein consists of two transmembrane domains, each comprising six membrane-spanning helices, and two intracellular ATP-binding regions (nucleotide-binding domains; NBDs). Phosphorylation sites (serine residues S661, S667, S671 and S683 [6]), depicted by green circles, are located within the linker region connecting the two domains. Glycosylation of P-gp occurs at the asparagine residues N91, N94 and N99 [6], depicted by branched lines between transmembrane helices 1 and 2. In the brain, P-gp is expressed on the luminal surface of BBB endothelial cells. Here, its mRNA and protein expression and activity levels are up- or downregulated by a range of transcription factors, miRNAs and post-translational mechanisms. The expression of P-gp protein has additionally been identified in pericytes, astrocytes [7,8], neurons [7,9] and microglia [10] of the brain.

P-gp is encoded by the *ABCB1 (MDR1)* gene in humans and the *Abcb1a* and *Abcb1b* gene isoforms in rodents. The regulation of P-gp is highly complex, affecting mRNA and protein levels as well as transporter activity. Although P-gp regulation is well studied in multi-drug resistance in cancer owing to its overexpression in many tumour cell lines [5], in this review we will highlight mechanisms relevant to P-gp regulation at the BBB (summarized in Figure 1). We furthermore address the viability of targeting P-gp regulation as a therapeutic strategy, by discussing the attempts that have been trialled thus far, and the potential complications that may arise from such approaches.

## 2. Transcriptional Regulation

Transcriptional regulation of the human *ABCB1* gene is complex, involving numerous signalling pathways and transcription factors that influence the conversion of DNA to mRNA. The transcription factors that have been implicated in the regulation of brain-expressed P-gp, as well as two key stimuli for altered P-gp transcription, namely inflammation and oxidative stress, are discussed below.

### 2.1. NF-κB

The nuclear factor kappa-light-chain-enhancer of activated B cells (NF-κB) family of transcription factors serve a critical role in mediating immune responses by inducing the expression of pro-inflammatory cytokines and chemokines [11,12]. An NF-κB element has been identified within the promoter region of the human *ABCB1* gene, and accordingly, NF-κB activation has been shown to upregulate *ABCB1* transcription and thus P-gp expression. In fact, it is considered a “master regulator” of *ABCB1* transcription due to its involvement as a downstream mediator of an array of stress-induced signals [13], including inflammation [14], oxidative stress [15], and epileptic seizures [16]. One such pathway involves pro-inflammatory cytokine-induced activation of protein kinase C (PKC). Briefly, tumour necrosis factor α (TNFα) exposure can lead to activation of nitric oxide synthase (NOS) and PKC isoform β2, which ultimately activates NF-κB to enhance *ABCB1* transcription at the BBB [14]. In another pathway, oxidative stress induced by exposure to sulforaphane was shown to promote the expression and transport activity of P-gp in rat brain capillaries via activation of the neuroprotective molecular sensor of oxidative stress, nuclear factor erythroid-derived 2-like 2 (Nrf2). Although Nrf2 itself is a ligand-activated transcription factor, it has been suggested that Nrf2-mediated P-gp upregulation occurs via an indirect manner, instead involving activation of NF-κB via p53 and p38 signalling [17]. Indeed, administration of Nrf2 ligands in murine models of traumatic brain injury is neuroprotective and helps to preserve BBB integrity [18]. A third pathway involves the upregulation of P-gp expression and activity at the BBB in response to epileptic seizures [19,20]. Excess release of the excitatory neurotransmitter, glutamate, signals through the ionotropic N-methyl-D-aspartate (NMDA) receptor to activate phospholipase A2, which releases arachidonic acid. Arachidonic acid is converted to prostaglandin E2 via cyclooxygenase-2 (COX-2), which then signals through a receptor, likely EP-1, to activate NF-κB and thereby increase P-gp expression [13,21]. Seizure-induced upregulation of P-gp can be attenuated by the COX-2-selective inhibitors, indomethacin and celecoxib [19,20].

NF-κB has also been shown to indirectly repress *ABCB1* transcription [22]. Amyloid-β (Aβ) peptides are the key constituent of amyloid plaques that accumulate in the brain in Alzheimer’s disease. P-gp is involved in the clearance of Aβ from the brain by actively exporting the peptides across the BBB [23,24]. Intriguingly, Aβ peptides have been reported to compromise P-gp expression both in vivo (in mice expressing five familial Alzheimer’s mutations, FXFAD) and in vitro (in murine brain endothelial cells, bEnd.3) [12], thus impeding its own clearance from the brain [25]. Park et al. suggested this could occur via receptor for advanced glycation end products (RAGE)-NF-κB-dependent signalling. Inhibition of the NF-κB pathway in bEnd.3 cells using BAY-11-7082, which blocks phosphorylation of the inhibitor protein IkBα, attenuated Aβ_42_-induced decrease in P-gp expression and luciferase activity. Furthermore, treatment of these cells with a neutralising antibody against RAGE also prevented the reduction in P-gp expression [12]. In a subsequent study, Chen et al. revealed the nuclear receptor, peroxisome proliferator activated receptor γ (PPARγ) (discussed below) as a downstream mediator of this pathway [26]. Together, these findings suggest that Aβ binds and activates RAGE, resulting in enhanced NF-κB-mediated downregulation of PPARγ, which thereby represses *ABCB1* gene transcription and P-gp expression [12,26].

### 2.2. Sp3

Specificity protein 3 (Sp3), a member of the Sp family of transcription factors, has been identified in human brain endothelial cells (hCMEC/D3). Gromnicova et al. demonstrated that Sp3 associates with the GC-box within the *ABCB1* promoter region [27]. Interestingly, it was noted that the interaction between Sp transcription factors and the *ABCB1* promoter region varied depending on cell type. Although both Sp1 and Sp3 are expressed in brain endothelium, Sp3 exhibited greater association with the *ABCB1* promoter in these cells. Contrastingly, Sp1 preferentially associated with the promoter region in human colon-derived epithelial cells (Caco-2). These results suggest the potential for selective/cell-specific regulation of P-gp expression [27]. Considering P-gp is constitutively expressed in a variety of tissues in the body, including the intestines, liver, placenta and kidney [28], this would be desirable for therapeutic purposes to develop approaches to target specific organs and reduce collateral effects. However, further studies are still required to confirm whether enhancing or inhibiting the binding of Sp3 to the *ABCB1* promoter results in changes to P-gp mRNA and protein levels.

### 2.3. TCF/LEF

Activation of the T-cell factor/lymphoid enhancer factor (TCF/LEF) transcription factor induces *ABCB1* expression [5]. TCF/LEF is a downstream target of the Wnt/β-catenin canonical signalling pathway, which serves important functions in the development and maintenance of the BBB [29,30]. Binding of Wnt ligands to the Frizzled receptor and LRP5/LRP6 co-receptors leads to inactivation of the glycogen synthase kinase-3 (GSK3) enzyme. This protects cytoplasmic β-catenin from destruction, enabling its accumulation and translocation from the cytosol to the nucleus where it binds to the TCF/LEF transcription factor to induce target gene transcription, among which includes *ABCB1* [30]. Several studies have demonstrated that modulating Wnt/β-catenin signalling can lead to up- or downregulation of P-gp mRNA (and hence protein) expression.

Activation of Wnt signalling using a pan-Wnt agonist, Wnt3a (Wnt ligand), or GSK3 inhibitors (lithium chloride, 6-bromoindirubin-3′-oxime) increased P-gp mRNA, protein and efflux activity in hCMEC/D3 cells. These effects were abolished upon treatment with ICRT-3, a small molecule inhibitor that disrupts the binding of β-catenin to the TCF-4 transcription factor. Similarly, treatment with Dickkopf-1 (Dkk-1), a naturally occurring peptide inhibitor of the Wnt/β-catenin pathway, or quercetin which inhibits TCF transcriptional activity, significantly decreased P-gp mRNA and protein expression [29,31]. Temozolomide, an anti-cancer drug used for the treatment of glioblastoma, was shown to downregulate P-gp mRNA and protein expression in hCMEC/D3 cells via disruption of Wnt/β-catenin signalling. This downregulation was accompanied by increased BBB permeability of P-gp substrates (rhodamine 123, doxorubicin and vinblastine) resulting from impaired P-gp transport activity. Mechanistically, temozolomide is able to methylate the promoter of the Wnt3 gene, thus reducing Wnt3 expression, leading to reduced β-catenin transcriptional activity [32].

### 2.4. Nuclear Receptors

Nuclear receptors are a family of ligand-activated transcription factors that bind directly to DNA to regulate the expression of target genes. A number of nuclear receptors have been shown to affect *ABCB1* transcription, as detailed below [33]. The associated ligands are wide-ranging, encompassing steroid hormones, oxysterols, vitamins, therapeutic drugs, and environmental toxins [3]. These ligands are able to cross the plasma membrane to directly bind with the cytosolic nuclear receptor, without first needing to interact with cell-surface receptors [34].

#### 2.4.1. Pregnane X Receptor (PXR) and Glucocorticoid Receptor (GR)

PXR is activated by a number of endogenous and xenobiotic ligands, including steroids, glucocorticoids, and various therapeutic drugs [4]. *ABCB1* transcription can be induced by PXR activation. Using DNA binding assays and transfections, Geick et al. identified an enhancer element within the *ABCB1* upstream region containing a cluster of DR4 response elements to which PXR, heterodimerised with RXRα, can bind to mediate *ABCB1* induction [35]. In vitro and ex vivo studies have corroborated these findings. Exposure of the PXR ligands, rifampin and hyperforin, was associated with significantly elevated P-gp mRNA, protein expression and transport activity in porcine brain capillary endothelial cells [36] and in brain capillaries isolated from transgenic mice expressing human PXR (hPXR) [37].

Several drugs and phytochemical compounds, including rifampicin, phenytoin, carbamazepine, doxycycline, hyperforin and curcumin, have been shown to induce P-gp expression via activation of PXR [38]. Some of these have been investigated for their potential clinical use. For example, in a mouse model of Alzheimer’s disease, St John’s wort extract containing 5% hyperforin increased cerebrovascular expression of P-gp protein and significantly reduced soluble Aβ_40_ and Aβ_42_ peptide concentrations and plaque load in the brain [39]. Correspondingly, administration of the endogenous steroid hormone pregnenolone, which is also a PXR ligand, into Alzheimer’s model mice expressing lower levels of P-gp than wild-type mice, restored BBB P-gp protein expression and transport activity, resulting in significantly reduced capillary membrane deposition of Aβ_40_ and Aβ_42_ peptides [23]. However, daily administration of rifampicin and/or doxycycline for 12 months did not yield any symptomatic improvements in human subjects with mild-to-moderate Alzheimer’s disease [40]. This was likely due to greater induction of P-gp expression in the periphery rather than at the BBB [41,42].

Many studies have also explored the effects of the synthetic glucocorticoid, dexamethasone, on P-gp expression. Treatment of brain endothelial cells from rats [43] or foetal guinea pigs [44] with dexamethasone dose-dependently and reversibly induced P-gp mRNA expression, protein expression, and transport activity. Dexamethasone, a known PXR ligand, was shown to upregulate PXR expression [43]. However, it was noted the aforementioned effects on P-gp upregulation were also significantly impaired (but not entirely inhibited) by RU486, a GR antagonist. Thus, dexamethasone appears to upregulate P-gp expression and function by acting via both PXR and GR [13,43]. Indeed, the presence of a glucocorticoid-responsive element has been identified in the murine *Abcb1b* promoter region [45].

#### 2.4.2. Liver X Receptor (LXR)

The liver X receptors LXRα and LXRβ play important roles in transcriptional regulation of genes implicated in lipid metabolism and cholesterol homeostasis. LXR is activated endogenously by oxysterols, which leads to the formation of a heterodimer with RXR and subsequent binding to LXR response elements within the promoter region of target genes to activate transcription [46]. Activation of LXR using the synthetic agonist T0901317 has been shown to enhance the expression of P-gp protein in mouse brain capillaries [47]. This finding was corroborated in an in vitro BBB model utilising bovine brain capillary endothelial cells (BCECs), wherein 24 h treatment with LXR ligands (the oxysterols 24S-hydroxycholesterol and 27-hydroxycholesterol, and T0901317) significantly upregulated the mRNA and protein expression of P-gp. This was accompanied by reduced cellular influx of the P-gp substrates [^3^H]-colchicine and soluble Aβ_40_ peptides. Thus, oxysterols appear to induce P-gp expression and activity via LXR activation, resulting in increased restriction of the influx of P-gp substrates across BCECs [48].

LXR activation not only increases P-gp expression, but is also separately involved in the regulation of apolipoprotein E expression (which affects several aspects of Aβ metabolism), neuroinflammation, and maintenance of BBB endothelial integrity [47,49,50]. Therefore, activation of the LXR pathway presents attractive potential as a therapeutic strategy in Alzheimer’s disease for enhancing P-gp activity, and alleviating the Aβ burden and its associated neurodegenerative effects [51,52], as well as in ischaemic strokes to help restore the BBB following hypoxia-induced BBB breakdown [47,53]. Nonetheless, an aspect requiring further consideration is that many LXR agonists, such as T0901317 and GW3965, activate both the LXRα and LXRβ isoforms to a comparable extent [54]. The two isoforms exhibit different expression patterns depending on cell type and serve differential physiological functions [55]. One study showed that LXRα, but not LXRβ, is critical for the maintenance of BBB integrity [55]. However, activation of LXRα is associated with increased hepatic lipogenesis which can increase susceptibility to atherosclerosis, leading to increased preference for the development of LXRβ-specific agonists [54,56,57].

#### 2.4.3. Peroxisome Proliferator Activated Receptor (PPAR)

PPARα, a master regulator of lipid metabolism that is activated by dietary lipids and xenobiotics, is also implicated in P-gp regulation. In vitro exposure of rat brain capillaries, as well as in vivo exposure of mouse brain to PPARα ligands (linoleic acid, clofibrate, or perfluor-oalkyl fire-fighting foam components) increased P-gp protein expression and efflux capacity at the BBB. These effects were abolished by the administration of GW6471, a specific PPARα antagonist [58].

#### 2.4.4. Constitutive Androstane Receptor (CAR)

P-gp expression is also positively regulated by CAR. Treatment of mouse brain capillaries with TCPOBOP (a mouse-specific CAR ligand), or rat brain capillaries with phenobarbital (CAR activator) significantly increased P-gp protein expression and activity, the latter of which was evidenced by enhanced luminal accumulation of the P-gp substrate, NBD-CSA. Activation of CAR requires dephosphorylation by protein phosphatase 2A (PPA2); accordingly, the effects of phenobarbital on P-gp were abolished by treatment with a PP2A inhibitor. Nevertheless, it remains to be confirmed whether CAR-mediated upregulation of P-gp occurs via direct transcriptional activation, or indirectly via CAR-induced signalling mechanisms [59].

#### 2.4.5. Aryl Hydrocarbon Receptor (AhR)

AhR ligands consist mainly of environmental pollutants such as polycyclic aromatic hydrocarbons, halogenated aromatic hydrocarbons and dioxins [60]. Treatment of rat brain capillaries with the AhR receptor ligands, TCDD and β-napthoflavone, enhanced P-gp protein expression and transport activity, and the effects of TCDD were counteracted by the AhR antagonists, α-napthoflavone and resveratrol, demonstrating that AhR activation positively regulates P-gp [61].

#### 2.4.6. Vitamin D Receptor (VDR)

Activation of VDR with the physiological ligand, 1α,25-dihydroxyvitamin D3, has been shown to upregulate P-gp expression and increase efflux of the P-gp substrate digoxin at the BBB in mice [62]. In subsequent studies by the same group, VDR activation-mediated induction of P-gp not only reversed accumulation of Aβ peptides in rat brain endothelial and human hCMEC/D3 cells [63], but also decreased cerebral Aβ deposition and improved conditioned fear memory in transgenic AD mice [64]. The VDR pathway has also been implicated in P-gp downregulation in Parkinson’s disease. In two separate mice models of Parkinson’s disease (6-hydroxydopamine-induced model, and α-synuclein preformed fibril injection model), transcriptional repression of the *VDR* gene and its downstream target gene, *MDR1a*, were restored upon treatment with 1α,25-dihydroxyvitamin D3. Treatment with this VDR ligand furthermore restored brain vascular endothelial expression of P-gp [65].

### 2.5. Inflammation

The effects of inflammation on P-gp are intricate and multifarious. Some studies have suggested inflammation increases P-gp expression and/or activity, whereas other studies have suggested the opposite (reviewed in [66]). For instance, treatment of RBE4 cerebral endothelial cells with TNFα induced P-gp mRNA (at 2–24 h) and protein expression (after 6–24 h of incubation), resulting in reduced cellular uptake of the P-gp substrate vinblastine [67]. Prolonged treatment of hCMEC/D3 cells with TNFα for 72 h similarly increased P-gp mRNA and protein expression, although P-gp-mediated transport of rhodamine 123 remained unchanged. In these cells, other pro-inflammatory cytokines, namely interleukin (IL)-1β and IL-6, slightly reduced P-gp mRNA, but had no effect on protein or activity levels [68]. Contrastingly, human U373MG glioblastoma cells transfected to express TNFα exhibited reduced P-gp protein expression, in a manner inversely proportional to the amount of TNFα secreted, which was accompanied by reductions in rhodamine 123 transport and increased chemosensitivity to vincristine and doxorubicin [69]. In guinea pig-derived brain endothelial cells, treatment with IL-1β, IL-6 and TNFα for 24 h reduced P-gp mRNA levels and transport function [70]. In vivo injection of lipopolysaccharide (LPS), which can induce the release of IL-1β, IL-2, IL-6, TNFα and interferon (IFN)-γ [71], into rat brain rapidly and significantly reduced P-gp mRNA levels and transport activity between 6–24 h [66].

While differences in the specific cytokines, concentrations of those cytokines, and cell lines employed across these experiments likely contributed to the varying effects observed, it appears that time of exposure to inflammatory mediators also has significant repercussions for P-gp [4]. In this regard, short-term exposure of isolated rat brain capillaries to TNFα rapidly and reversibly reduced the luminal accumulation of NBD-CSA (a fluorescent P-gp substrate). The inhibitory effect of TNFα was achieved within minutes and persisted for up to four hours, and was of comparable magnitude to that of PSC833, a potent P-gp inhibitor. The authors proposed the following mechanistic pathway: TNFα acts through the TNF-R1 receptor to release endothelin (ET)-1, which activates ET_B_ receptors, resulting in activation of NOS and then PKC isoform β1, which subsequently reduces P-gp transport activity without affecting its expression (further discussed in “Indirect Regulation”) [72,73]. The same group later demonstrated that a longer-term exposure to TNFα of 6 h increased both P-gp protein expression and transport activity in rat brain capillaries. Similar to before, TNFα activates TNF-R1, releasing ET-1, which binds ET_B_ to activate NOS. However, the longer-term exposure subsequently activates PKC isoform β2, which activates the NF-κB transcription factor to enhance P-gp levels [13,14]. It could be hypothesized that this upregulation of P-gp serves as a compensatory strategy to protect the CNS when exposed to prolonged inflammation [14,74], but further investigations are needed before any conclusions can be made.

Neuroinflammation is associated with many neurological and neurodegenerative conditions, including Alzheimer’s disease, Parkinson’s disease, HIV-associated dementia, multiple sclerosis and amyotrophic lateral sclerosis (ALS), in which it is considered to be both a consequence as well as driver of pathology [75,76,77]. Studies that have thus far examined the effects of inflammatory mediators on P-gp expression in the brain have largely been limited to in vitro and animal models with relatively short periods of exposure to inflammation (<72 h). It thus remains uncertain how P-gp in the brain may be affected in situations of persistent long-term exposure to inflammatory mediators that occur in chronic neuroinflammatory conditions.

### 2.6. Oxidative Stress

Glutathione is an important and ubiquitous tripeptide that functions as an antioxidant to protect cells from oxidative stress. Glutathione depletion, which has been implicated in neurological disease, leaves cells more vulnerable to oxidative stress, and has been demonstrated to upregulate P-gp mRNA and protein expression as well as transport activity in vivo [78] and in vitro [79] at the rat BBB. These changes were reversed upon treatment with the reactive oxygen species (ROS) scavenger, *N*-acetylcysteine, thus implicating oxidative stress in the regulation of P-gp [78,79]. Hydrogen peroxide-induced oxidative stress has similarly been shown to invoke concentration-dependent increases in P-gp expression and activity in vitro via induction of NF-κB signalling and transcription [15,80]. Air pollution, including diesel exhaust particles, can promote oxidative stress by activating NADPH oxidase, thus increasing the production of the pro-inflammatory cytokine, TNFα. Correspondingly, exposure of isolated rat brain capillaries to such pollutant particles for 6 h induces P-gp expression and activity. However, unlike the LPS-induced pro-inflammatory context [14], this occurs via a mechanism independent of NF-κB activation. Instead, oxidative stress-induced TNFα production activates the c-Jun amino-terminal kinase (JNK) pathway of mitogen-activated protein kinase (MAPK) signalling, to activate the activator protein 1 (AP-1) transcription factor, to promote *ABCB1* transcription [81]. Based on the experimental data available, which reflect exposure periods up to 24 h, P-gp at the BBB is upregulated, likely as a compensatory protective measure, in response to oxidative stress. However, the effect of chronic exposure to oxidative stress, which may bear greater relevance in neurological disease states, on P-gp expression remains unknown.

## 3. Post-Transcriptional Regulation

MicroRNAs (miRs) are small single-stranded non-coding RNA molecules ranging from 19–25 nucleotides in length that can post-transcriptionally regulate mRNA expression. Several miRs have been shown to bind to the 3′ untranslated region (3′UTR) of *ABCB1* mRNA, resulting in suppression of protein expression via mRNA degradation or inhibition of translation [82]. For instance, miR-298 binds directly to *ABCB1* to inhibit P-gp expression, leading to reduced efflux of anti-epileptic drugs from drug-resistant human brain microvascular endothelial cells and U87-MG glioblastoma cells [83]. Similarly, miR-451, miR-331-5p, miR-298, miR-145 and miR-27a have been shown to decrease P-gp expression, thus facilitating the reversal of MDR in cancer cell lines [5,82]. MiRs, including miR-146a-5p and miR-138, can also regulate P-gp expression indirectly, by suppressing the expression of transcription factors such as NF-κB/p65, that control *ABCB1* transcription [84,85]. Thus far, such studies investigating the regulation of P-gp by miRs have only been conducted in vitro, hence their physiological relevance remain to be examined in the in vivo setting.

## 4. Post-Translational Regulation

The addition (or removal) of modifying groups to a protein following its biosynthesis augments the complexity and diversity of protein expression and function beyond what is encoded by the genome. Post-translational modifications to P-gp include phosphorylation, glycosylation, and ubiquitination, which have been shown to affect the stability and activity of the protein.

### 4.1. Phosphorylation and Glycosylation

Phosphorylation and glycosylation are both major types of post-translational modifications. Although they are not unique to brain-expressed P-gp, they are nevertheless included here for completeness.

Human P-gp is phosphorylated by protein kinases A (PKA) and C (PKC) (Figure 1), contributing to the regulation of transport activity [72,73,86,87]. P-gp also contains a consensus sequence for phosphorylation by the serine/threonine protein kinase, Pim-1. Pim-1-mediated phosphorylation protects under-glycosylated P-gp protein from degradation by proteases in the endoplasmic reticulum, before it undergoes further glycosylation within the Golgi [88].

In the Golgi, *N*-linked glycosylation of P-gp occurs at three asparagine residues located on the first extracellular loop of the protein (Figure 1). This yields a mature fully glycosylated species with an apparent molecular weight of 170 kDa [6]. Glycosylation is important for stability and trafficking of the protein to the plasma membrane, however it is not essential for P-gp transport function [89,90].

### 4.2. Ubiquitination

Ubiquitination refers to the ligation of ubiquitin, a 76 amino acid polypeptide, to a target protein, via a process involving the sequential actions of three enzymes—ubiquitin-activating enzyme (E1), ubiquitin conjugating enzyme (E2), and ubiquitin ligase (E3) [91]. Ubiquitinated proteins may be targeted for degradation via the 26S proteasome (ubiquitin-proteasome system; UPS) or via the autophagy-lysosome pathway, or may undergo non-proteolytic processes such as endocytosis or recycling which influence the level of active protein available [92].

Ubiquitinated P-gp has been demonstrated to undergo degradation via the UPS. Immunoprecipitation experiments indicate the existence of ubiquitinated P-gp in various drug-resistant and *ABCB1*-transfected cell lines, and transfection of the latter with ubiquitin significantly increased the levels of ubiquitinated P-gp. This concomitantly reduced the overall levels of P-gp protein expression, resulting in enhanced intracellular accumulation of the P-gp substrate doxorubicin [90]. In mice, chemical inhibition of the E1 enzyme by PYR-41 has been shown to prevent ubiquitination of P-gp, thereby preventing the degradation of P-gp protein and preserving its transport activity at the BBB [93,94]. Inhibition of proteasomal activity using MG-132, lactacystin and bortezomib rapidly increases the accumulation of ubiquitinated P-gp, indicating that degradation of P-gp occurs in the proteasome [90,94,95]. Inhibiting UPS-mediated degradation of P-gp could thus serve to restrain the downregulation of P-gp activity observed in ageing and disease. Compounds targeting different components of the ubiquitin-proteasome pathway, including proteasome inhibitors, E1/E2/E3 ligase modulators, and deubiquitinase inhibitors, are already employed in the oncology setting [96,97]. However, targeting of the E3 ligase may be favourable due to is greater substrate specificity as compared with E1 and E2 ligases and proteasomes [92]. Although several E3 ligases have been identified that either recognise P-gp as a substrate [95] or indirectly regulate [98,99,100] P-gp in the context of cancer, only one E3 ligase, namely neural precursor cell-expressed developmentally downregulated protein 4-1 (NEDD4-1), has thus far been investigated with respect to P-gp expression in the brain. In brain capillaries isolated from mice, P-gp protein expression is inversely correlated with protein expression of Nedd4 (the rodent homologue of NEDD4-1) [101]. In vitro experiments demonstrate that NEDD4-1 recognises P-gp as a substrate, leading to its ubiquitination and internalisation from the cell surface [101]. Correspondingly, knockdown of Nedd4 using siRNA increases P-gp protein expression and transport activity [102]. Interestingly, exposure of mouse brain capillaries to human Aβ_40_ peptides enhances Nedd4 protein expression and concomitantly decreases P-gp protein expression [101], thus paving the potential for modulation of NEDD4-1 as a strategy to restore P-gp expression and activity in Alzheimer’s disease.

## 5. Indirect Regulation

P-gp can also be indirectly regulated via mechanisms that influence the activity of the protein in an acute and reversible manner without impacting its expression levels. For instance, many pharmacological compounds can inhibit P-gp activity by acting as competitive substrates for transport [103,104]. Other mechanisms identified involve sphingolipid signalling, vascular endothelial growth factor (VEGF) signalling, trafficking and internalisation, and alterations to the membrane lipid environment.

Sphingolipid signalling can differentially modulate P-gp transport activity, without affecting protein expression. On one hand, sphingosine and fingolimod (a sphinogsine-1-phosphate analogue) were found to rapidly and reversibly reduce P-gp export activity in isolated rat brain capillaries [105]. Short-term exposure of these brain capillaries to TNFα led to downstream activation of PKCβ1, and sphingosine signalling through sphingosine-1-phosphate receptor 1 (S1PR1) was found to be responsible for mediating the effect of PKCβ1 activation on inhibiting P-gp activity [105]. On the other hand, a different sphingolipid, ceramide 1-phosphate (C1P), was found to increase P-gp transport activity at the BBB. This occurred via a separate pathway implicating the COX-2/prostaglandin E2 signalling cascade, which was suggested to promote the trafficking of intracellularly located P-gp to the plasma membrane surface to enhance activity without altering overall protein expression levels [106].

Vascular endothelial growth factor (VEGF) is a signalling protein that stimulates vasculo- and angio-genesis. Its overexpression in the brain has been associated with neurological disease, brain injury and BBB dysfunction [107]. In vitro experiments utilising isolated rat brain capillaries, as well as in situ rat brain perfusion studies, collectively demonstrate that exposure to VEGF leads to rapid and reversible reductions in P-gp transport activity, without affecting protein expression [107]. VEGF signals through flk-1 and Src kinases to move P-gp protein from the plasma membrane to a sub-apical or vesicular compartments, rendering it unable to contribute to membrane efflux activity. Furthermore, VEGF increases Tyr-14 phosphorylation of the caveolae protein, caveolin-1, by Src kinase, which has been demonstrated to reduce P-gp transport activity in rat brain endothelial cells, providing an additional mechanism for the downregulation of P-gp function [107,108].

Considering that P-gp function is dependent upon the expression of the protein on the cell surface, preventing its internalisation can enhance its activity. Nocodazole is a microtubule inhibitor that disrupts the intracellular trafficking and blocks internalisation of membrane proteins. Treatment of a mouse model of Alzheimer’s disease with nocodazole not only restored P-gp protein expression levels to that of wild-type mice and enhanced P-gp transport activity, but also lowered overall human Aβ_40_ and Aβ_42_ levels in the brain [109].

Finally, the membrane environment, including characteristics relating to lipid composition and fluidity, within which P-gp is localized can also affect its function. Lipid rafts refer to membrane microdomains that are more highly enriched with cholesterol and glycosphingolipids, display greater order, and are more tightly packed than the surrounding bulk membrane [110]. P-gp exists in both raft and non-raft membrane domains, and this may consequently affect its stability, ATPase activity, substrate binding and transport function [111]. For instance, cholesterol depletion of cell membranes has been reported to decrease substrate binding and P-gp transport function, whereas presence of cholesterol stimulates basal ATPase activity of P-gp [111,112]. Furthermore, the effect of cholesterol on substrate-dependent P-gp ATPase activity has been suggested to vary depending on the size of the transported substrate, with greater stimulation associated with smaller substrates [113].

## 6. Significance and Implications of Modulating P-gp

As a “gatekeeper” of the BBB, P-gp serves a crucial role in protecting the CNS by mediating selective extrusion from, and impeding entry of substrates into the brain [2]. Disruptions to BBB function resulting from increased barrier permeability and transport protein dysfunction expose the vulnerable environment of the CNS to environmental toxins and build-up of metabolic waste which can drive pathology [114]. Indeed, reduced P-gp expression and compromised P-gp function at the BBB have been implicated in the pathogenesis of neurodegenerative conditions including Parkinson’s disease [65,115,116], Alzheimer’s disease [25,117] and multiple sclerosis [118], as well as with ageing [119,120,121]. Upregulating P-gp activity could be a viable strategy for restoring P-gp function that has been impaired as a result of disease and/or ageing. As discussed in this review, strategies such as increasing protein transcription, enhancing protein trafficking and stability, and curtailing protein degradation, have been provisionally investigated with respect to their therapeutic potential. However, while these strategies may indeed enhance P-gp expression and transport activity, further investigations are warranted and important caveats must be taken into consideration when assessing their clinical viability.

In addition to the brain, P-gp is expressed throughout the periphery [28]. Consequently, it is important to consider the potential impact of modulating P-gp activity on the normal physiological functions of the transporter at these sites. Reduced P-gp activity is anticipated to elicit greater vulnerability to toxic substances, whereas increased P-gp activity may impart greater protection. However, considering the large and diverse array of substances known to be transported by P-gp [104], the latter may occur at the expense of impaired tissue penetration of therapeutic drugs [122]. In CNS conditions such as epilepsy [117,123,124], ALS [125], and brain tumours [3], P-gp expression and activity in the brain have been found to be upregulated as a consequence of disease, thereby contributing to pharmacotherapeutic failure since many drugs used to treat these conditions are substrates of P-gp. On the other hand, intentional manipulation of P-gp activity has been investigated as a therapeutic approach to improve drug delivery to the brain, and indeed has shown promise in in vitro and animal models [105,126,127,128]. However, these strategies have not been clinically adopted as of yet [129]. Ultimately, care must be exercised in individuals experiencing polypharmacy and multi-morbidities with respect to the potential for drug–drug and drug-disease interactions involving P-gp.

Several avenues of research are deserving of further investigation. Firstly, although the present review has predominantly focused on P-gp expressed at the BBB, expression of P-gp has additionally been reported in other brain cells including pericytes, astrocytes [7,8], neurons [7,9] and microglia [10] (Figure 1). The regulation of P-gp in these cells is presently unknown, but could bear pathophysiological significance considering that dysfunction of these cells has been implicated in neurodegenerative diseases [130,131,132]. Secondly, there remains the possibility that P-gp could be differentially regulated depending on its localisation, for example in the CNS versus the periphery [27]. Lastly, while there are many effective strategies to upregulate P-gp activity, in vivo studies are warranted to establish whether these translate into improved clinical outcomes.

Elucidating the mechanisms by which P-gp is regulated not only helps us understand its functional role in health and disease, but may also facilitate the development of novel and nuanced strategies to manipulate P-gp for therapeutic benefit. Rather than utilising a global/non-specific approach to modulating P-gp activity, targeting mechanisms of P-gp regulation that are specifically implicated in disease pathophysiology can offer a more effective approach while also limiting the potential for adverse collateral effects.

## Data Availability

Not applicable.
